# From crisis to resilience: strengthening climate action in OECD countries through environmental policy and energy transition

**DOI:** 10.1007/s11356-023-29970-z

**Published:** 2023-10-26

**Authors:** Rui Ma, Nabila Abid, Suchang Yang, Fayyaz Ahmad

**Affiliations:** 1 Present Address: School of Economics, Lanzhou University, Lanzhou, 730000 Gansu China; 2 Department of Management and Business Administration, University “G. d’Annunzio” of Chieti-Pescara, Chieti, Italy

**Keywords:** Environmental policy, Energy transition, Climate change, CS-ARDL, OECD

## Abstract

Climate change represents a grave challenge to the global economy, environment, and societal well-being, jeopardizing their long-term sustainability. In response to this urgent issue, the study emphasizes the significance of environmental policy and energy transitions as fundamental factors in addressing the climate change crisis. The research draws upon data from OECD countries spanning the period between 1990 and 2020, utilizing robust econometric techniques to assess data properties. The study utilizes a comprehensive CS-ARDL model, incorporating multiple control variables like non-renewable energy GDP, foreign direct investment (FDI), and research and development (R&D). The results show that environmental policy and energy transitions are effective in reducing climate change impacts in the form of CO_2_ emissions. The non-environmental factors like GDP and FDI are positively associated and thereby accelerate climate change processes, whereas R&D promotes environmental protection by reducing CO_2_ emissions. Based on these findings, the study advocates for the implementation of rigorous policy measures by OECD economies to strengthen and enforce environmental policies to ensure compliance and foster sustainable practices across sectors. The study also suggests that OECD must promote energy transitions by investing in renewable energy sources at the mass level (micro and macro) and phasing out reliance on non-renewable energy.

## Introduction

The problem of climate change and global warming has become a significant and urgent concern in the twenty-first century (UNEP 2019). The proliferation of harmful gases from increased human activities has led to substantial environmental pollution, posing significant global environmental challenges. In response, various environmental initiatives have been implemented over the past two decades with the primary objective of facilitating the energy transition to facilitate low-carbon economies. Such initiatives aim to mitigate the detrimental climate change impacts (Abid et al. 2023a; Javed et al. 2023a; Owjimehr and Samadi 2023). In recent decades, environmental policies have gained a significant grip and have become prominent focal points in the strategic agendas of governments and international authorities to combat climate change. This heightened emphasis is evident in joint initiatives like the 2015 Paris Agreement, which exemplifies ambitious measures to combat climate change. The rigorous activities to curb emissions to mitigate climate change necessitate the implementation of robust policies and regulatory frameworks that prioritize environmental protection. The initiative through the Paris Agreement and Kyoto Protocols entails the establishment of regulatory frameworks and environmental policies specifically designed to address the challenges posed by climate change and minimize its adverse impacts (Sarkodie and Strezov 2019; Sezgin et al. 2021). The significance of environmental policies at the national and international levels has become increasingly important in successfully tackling pollution, resource depletion, and escalating carbon emissions (Espoir et al. 2022).

The pathway to effectively address environmental crises relies heavily on the policy objectives set forth by various governments and international authorities and their strategic policy decisions, which play a crucial role in shaping environmental outcomes (Dechezleprêtre et al. 2011; Wenbo and Yan 2018). By adhering to these strategies, nations can effectively curb harmful emissions and enhance energy efficiency, fostering sustainable development and resilience in the face of global environmental challenges (Chen et al. 2022). Hille et al. (2020) posited that to maximize the advantages derived from environmental policies; it is imperative to implement an approach over a prolonged duration, thereby demonstrating a commendable level of regulatory consistency in the realm of environmental governance. The widespread concern surrounding climate change made policymakers devises stringency policies and focus on the formidable task of reforming the world’s energy system (Loorbach et al. 2017).

**Fig. 1:**
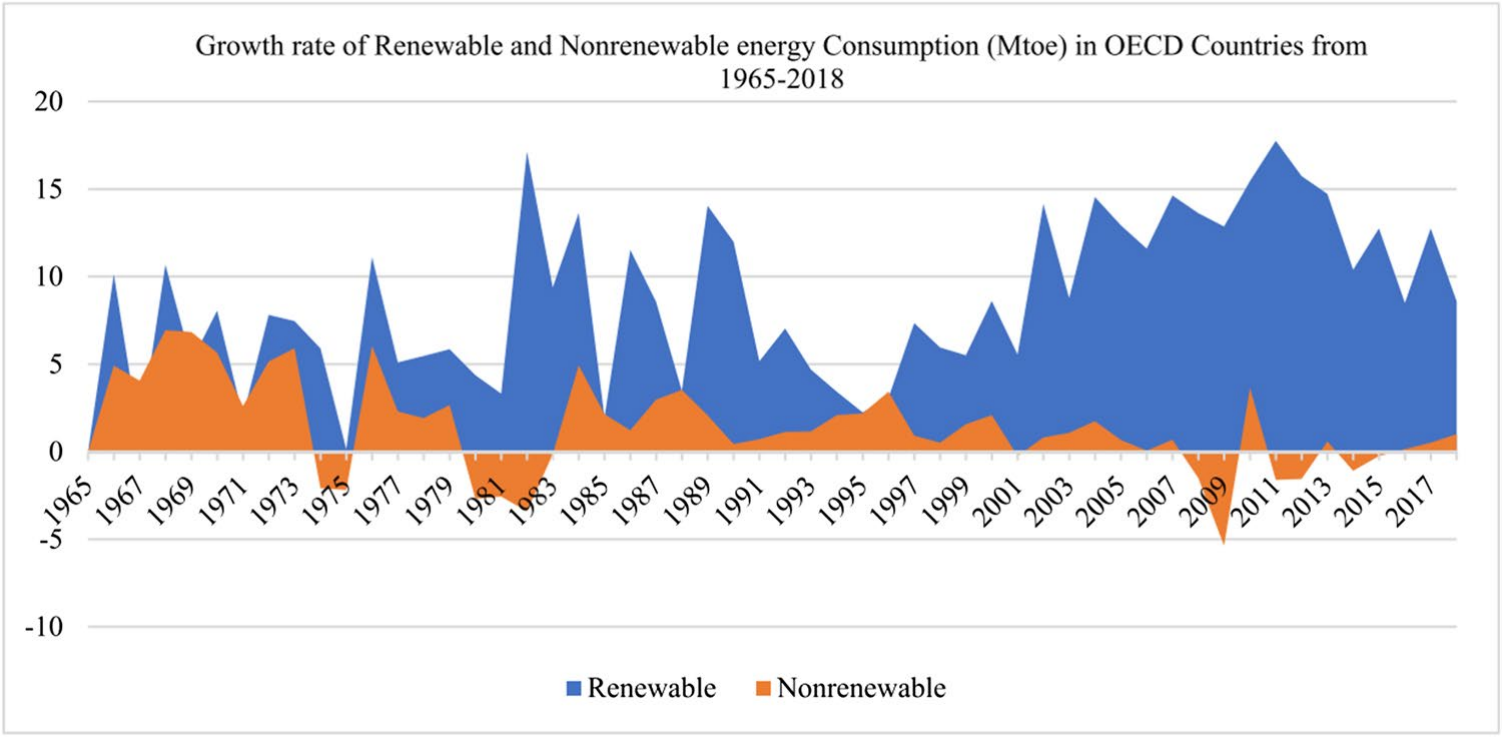
Renewable energy consumption pattern in OECD (OECD 2021)

The urgent need for sustainable and environmentally friendly power sources has emerged as a top priority within the global policymaking framework, driven by the severe repercussions of excessive reliance on fossil fuels, which result in the emission of carbon emissions and the imminent depletion of natural resources (IPCC 2021). Consequently, numerous countries have prioritized the promotion of renewable energy production to foster low-carbon productivity (Jabeen et al. 2023; Javed et al. 2023c; Liang et al. 2023). The notion of the energy transition has consistently emerged as a prominent solution in the discourse surrounding climate change within numerous UN climate change negotiations. It is a prerequisite for attaining the ambitious 2.0 °C target set forth by the agreement. Transitioning from conventional energy consumption patterns to adopting clean and sustainable energy sources is widely acknowledged as a pivotal component of achieving sustainable development objectives (Owjimehr and Samadi 2023). Encouraging statistics indicate a notable decline in carbon emissions in Fig. 1. In terms of carbon emissions, the deceleration observed in 2021 only marginally meets the annual average slowdown required to fulfill the climate targets outlined in the Paris Agreement within the next three decades on a global scale (Irfan et al. 2023). The upward trend in the utilization of renewable energy sources, as illustrated in Fig. 1, is an encouraging development in the global energy landscape. From 2010 to 2018, the share of renewable energy in final energy consumption gradually rose, growing from 16.4 to 17.1% (United Nations 2022). However, it is essential to acknowledge that the advancements in securing energy access have been uneven and disparate among different regions worldwide. Therefore, it is evident that the journey toward a complete energy transition is still distant and requires concerted efforts on a global scale (Wei et al. 2023). The energy transition is imperative to approach the issue of environmental degradation and protection through a multifaceted and dynamic lens. While many developed countries have implemented environmental policies to preserve ecological integrity, paradoxically, these nations have historically exhibited the highest levels of carbon dioxide emissions per capita and continue to do so (Hansen et al. 2018; Razzaq et al. 2023).

**Fig. 2:**
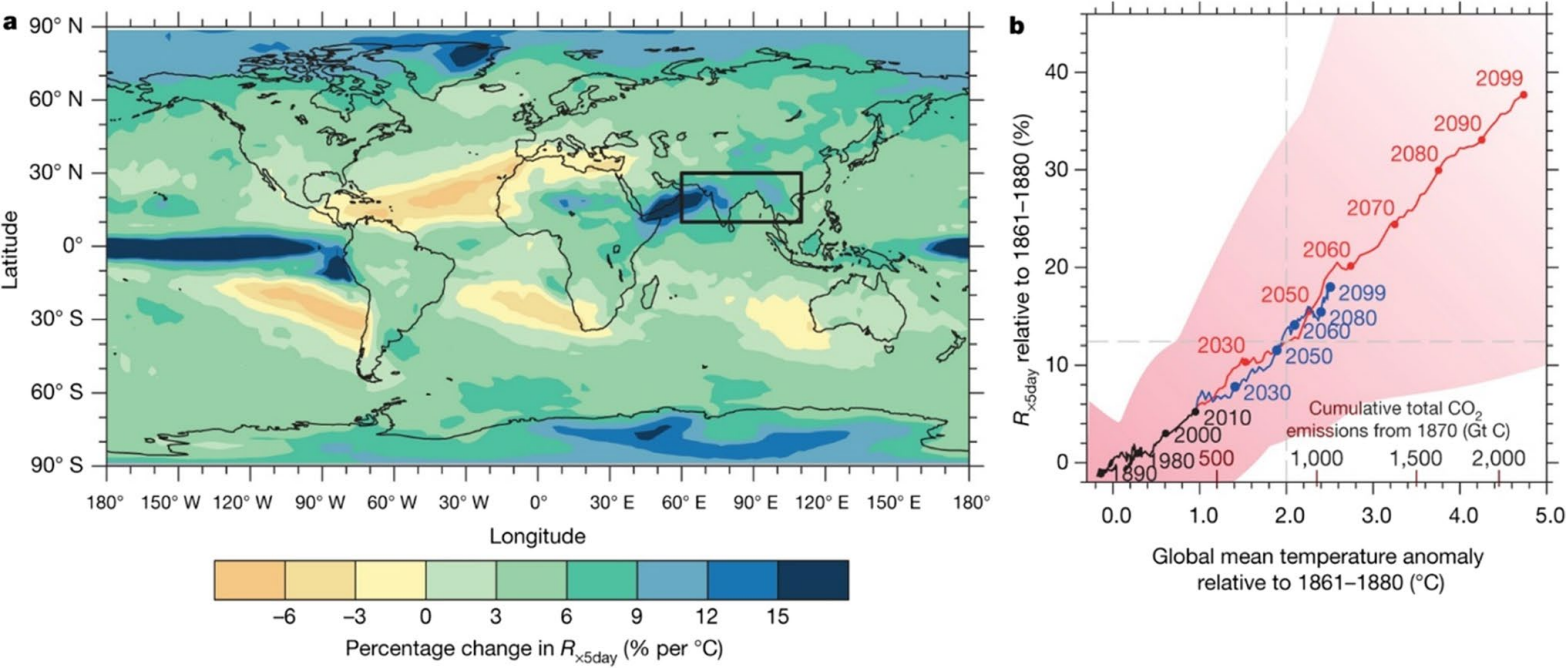
Scaling the magnitude of 5-day heavy precipitation events in relation to global mean temperature variations in conjunction with corresponding targets for cumulative CO_2_ emissions at a global level (Seneviratne et al. 2016)

This study primarily concentrates on investigating the OECD countries, given their significant reliance on non-renewable energy sources, resulting in substantial CO_2_ emissions increase. The OECD countries contribute approximately 36% of global non-renewable energy emissions (Fig. 2). Within these nations, industries dependent on energy contribute to approximately 29% of worldwide emissions, primarily due to their reliance on non-renewable resources (OECD 2021). It is imperative to highlight that the selected countries in this study are confronted with critical environmental challenges, resulting in unforeseen consequences within the ecological system that severely threaten climate change goals. The existing body of literature exhibits two notable limitations. The central focus of this discussion revolves around developing nations, highlighting their disproportionate vulnerability to the effects of climate change. These countries are expected to endure the greatest burden of climate change consequences (Mertz et al. 2009; Adenle et al. 2015). Nath and Behera (2011) and Stern (2006) have asserted the importance of exploring various contexts beyond developing nations to gain a holistic understanding of climate change. These studies’ results highlight that, despite achieving substantial economic growth, developed countries are not exempt from the consequences of climate change. As a result, it is critical to perform comprehensive research that includes both emerging and industrialized economies, as these countries engage in activities with major environmental repercussions (Simaens and Koster 2013; Sprengel and Busch 2011; Su and Moaniba 2017). One notable limitation observed in the existing body of literature is the excessive emphasis on organizational-level analysis (Ben Youssef et al. 2018; Costantini and Mazzanti 2012; Fabrizi et al. 2018), neglecting broader aspects. The purpose of this study is to fill this research gap by investigating the effectiveness of environmental policies and energy transition in reducing climate change in OECD countries. The research specifically tries to address the following research question: To what extent do environmental policies and energy transition contribute effectively to the mitigation of climate change in OECD nations? Following the insights presented by Dogru et al. (2019), this study proposes an analytical framework encompassing a comprehensive set of control variables. It acknowledges the significance of environmental factors and various non-climatic variables in determining the effectiveness and outcomes of climate change mitigation efforts. By integrating these additional variables, the proposed framework aims to provide a more comprehensive understanding of the complex mechanisms involved in climate change mitigation. Using the data from 1990 to 2020, the study has used extensive econometrics techniques to examine the underlying relationship between study variables. This research paper presents a comprehensive analysis aimed at enhancing the comprehension of the role of environmental policy and energy transition in addressing the complex challenges posed by climate change. By synthesizing existing evidence and incorporating robust empirical foundations, this study offers novel insights into the dynamics specific to OECD countries, with potential extrapolation to similar contexts. Furthermore, this paper lays the groundwork for future investigations, inviting alternative perspectives and levels of analysis to dissect this phenomenon further.

The following sections of this paper are organized as follows, offering comprehensive insights into the research conducted. The second section critically examines the existing literature, elucidating the evolution of the study parameters and establishing the intricate relationship between environmental policy, energy transition, and climate change. The third section outlines the methodology, providing a review of the methodological approach and data collection procedures. Subsequently, in the fourth section, the obtained results are comprehensively analyzed and interpreted. Finally, the fifth section presents the concluding remarks, along with a discussion of future perspectives for further exploration and advancement in this field of research.

## Literature review

The formidable challenge posed by climate change and its far-reaching consequences necessitates immediate and concerted efforts toward achieving sustainable development (Nations 2015). Consequently, scholars and policymakers have shown significant interest in comprehensively understanding the effect of human actions on the ecological order that has spurred the climate change process worldwide. Within this context, carbon dioxide (CO_2_) prominently emerges as the primary indicator of environmental deterioration speeding up climate change, owing to its status as the predominant greenhouse gas (GHG) emission (Solarin and Bello 2018). The efficacy of environmental policies in mitigating carbon dioxide (CO_2_) emissions has been the subject of investigation among a select group of researchers. According to the majority of these experts, the OECD’s environmental policy stringency index (OECD 2021) serves as a reliable proxy for assessing the strength and efficacy of environmental policy. According to Botta and Kózluk (2014), the environmental policy stringency index should serve as an initial and tangible step toward internationally measuring the degree of environmental policy over a significant period. Wolde-Rafael and Mulat-weldemeskel (2021) reveals that environmental policy stringency has come out as a crucial policy instrument in combating environmental degradation. By analyzing data from 1990 to 2012, Ahmed and Ahmed (2018) conducted estimations on China’s CO_2_ emissions until 2022, demonstrating that stringent environmental policies can help reduce emissions.

Moreover, recent research conducted by Ahmed (2020) found that regulations pertaining to the environment have an important part in supporting an overview of ecologically friendly innovations, resulting in a large reduction in CO_2_ emissions. Wang et al. (2020) undertook a study that demonstrated the negative influence of severe ecological strategies on CO_2_ levels. The research results of this investigation underline the need for stringent environmental policies. In a separate empirical analysis, Wolde-Rufael and Weldemeskel (2020) investigated the influence of conservation policy inflexibility on CO_2_ emissions. Their study covered the period from 1993 to 2014, and findings revealed a strong correlation between CO_2_ emissions and the level of environmental strategy stringency within the selected countries. Building upon their previous work, Wolde-Rafael and Mulat-weldemeskel (2021) conducted a follow-up study on the influence of environmental policy rigor on CO_2_ emissions in seven emerging nations. The study used an enhanced mean group estimator and spanned the years 1994 to 2015. The researchers discovered emissions and policy relationship, showing a nonlinear relationship between these factors. Moreover, the analysis unveiled a one-way causality, with both the environmental policy stringency significantly influencing CO_2_ emissions. Albrizio et al. (2017) investigated increasingly stringent environmental policies over the last two decades in a separate analysis. However, their research revealed that stricter environmental policies have limited long-term impacts on overall productivity, with their effects primarily manifesting in a short period. Bieth (2021) undertook a study to examine the control of financial progress on CO_2_ emissions in six Asian nations. The study established that economic expansion considerably impacts CO_2_ emissions within the economies investigated.

The shift toward sustainable energy on a universal gage is predominantly motivated by the urgency to tackle climate change and minimize the harmful impacts of air pollution at the local level. Energy consumption plays a central role in generating a significant portion of carbon dioxide emissions, underscoring the importance of prioritizing this domain. Around 66% of worldwide harmful emissions can be attributed to burning fossil fuels (Onoda 2009). The primary objective of the Paris Climate Agreement is to restrict the global temperature rise to less than 2 °C. This objective necessitates the complete elimination of carbon dioxide emissions from energy generation within the next five decades. More rapid reductions in emissions are imperative to achieve an even more ambitious target of limiting temperature increase to 1.5 °C. The transition to sustainable energy relies on two fundamental aspects: improving energy efficiency and substantially augmenting the utilization of renewable energy sources. However, the adoption and feasibility of renewable energy vary across countries (IRENA 2019; Javed et al. 2023b; Zhang et al. 2022). However, all economies must substantially augment their reliance on renewable energy. To accomplish this, it is crucial to accelerate improvements in energy intensity, targeting an annual increase from the current rate of 1.8% to as high as 2.8% by 2030. This objective aligns with the energy efficiency target of Sustainable Development Goal 7. Furthermore, these efforts must be sustained beyond 2030 and continue until 2050 (Energy Transitions Commission 2017; Gielen et al. 2019).

OECD countries wield tremendous economic power and have played a critical role in initiating and investigating climate change mitigation solutions within the framework of the United Nations (OECD 2017). These developed nations were expected to lead the way in sustainable development and economic growth, benefiting from their capacity to invest in cleaner technologies. However, recent assessments of carbon emissions reveal a troubling rise in emissions from OECD countries (OECD 2019). This challenges the notion that developed nations, which generally have higher levels of literacy, would exhibit greater environmental awareness among their populations (Emiru and Waktola 2018). As a result, it becomes crucial for policymakers within OECD countries to devise clean energy policies and environmentally friendly measures to tackle the challenges posed by climate change. Polzin et al. (2015) investigate the impact of government policies on renewable energy development. Furthermore, policy directives emphasize the importance of governmental financing in renewable energy industries while urging OECD countries to introduce monetary incentives to draw such investment. Jebli et al. (2016) highlight the significance of expanding international trade and increasing the adoption of renewable energy as an alternative energy source that serves as an effective climate change mitigation approach. This reasoning stems from the fact that developed countries tend to employ cleaner technologies in their international trade activities, gradually reducing carbon emissions over time. In another study, Shafiei and Salim (2014) explored the numerous factors influencing carbon emissions from 1980 to 2011. The researchers revealed that the increased use of fossil fuels contributes significantly to the rise in carbon emissions. In contrast, increased use of renewable energy sources results in lower energy usage. These findings imply that in order to address climate change effectively, it is critical to stimulate the widespread adoption of renewable energy sources.

In a study conducted by Zhu et al. (2016), the emphasis was on examining the relationship between FDI, energy consumption, economic growth, and carbon emissions in ASEAN countries. The study’s findings revealed that FDI aids in the reduction of carbon emissions. This shows that foreign investments in these nations can positively impact environmental sustainability by encouraging cleaner and more efficient practices. Furthermore, the study discovered that economic expansion has a detrimental impact on environmental quality in nations with high emissions. This means that rapid economic expansion in the absence of adequate environmental safeguards might result in increased carbon emissions and ecological damage. These findings emphasize the significance of implementing sustainable development techniques in order to balance economic growth and environmental preservation. In order to examine the relationships among nuclear energy, CO_2_ emissions, economic growth, and renewable energy from 1984 to 2007, Apergis and Payne (2010) investigated the correlation between CO_2_ emissions and renewable energy in a sample of developed and developing nations. Their investigation produced interesting findings. They discovered that, in the short run, CO_2_ emissions are not significantly decreased by using renewable energy sources. However, the study demonstrated that using renewable energy significantly reduces CO2 emissions over the long term. These results imply that although renewable energy may not have a substantial immediate influence on CO_2_ emissions, it does so over time. This demonstrates how crucial it is to make long-term commitments and investments in renewable energy sources in order to significantly reduce greenhouse gas emissions. Countries can endeavor to mitigate the consequences of climate change and achieve a more sustainable future by transitioning toward renewable energy on a bigger scale and encouraging its adoption globally. In a different study, Farhani (2013) looked into the connection between CO_2_ emissions, economic growth, and the use of renewable energy in MENA nations. The empirical results showed that there was no short-term causal relationship between these variables, with the exception of unidirectional causality. However, over time, the results showed a one-way relationship between economic growth (GDP) and CO_2_ emissions, and renewable energy consumption. Additionally, Zoundi (2017) showed that renewable energy had a detrimental effect on CO_2_ emissions. This effect has a growing long-term impact and shows that renewable energy is a competitive option to conventional fossil fuel energy for halting environmental damage.

## Econometric modeling

### Data description

We built a framework constructed on current research and applied an experiential technique supported by fiscal modeling to explore the impact of environmental policies and energy transition on climate change. The initial phase involves gathering relevant data on the variables of interest from diverse sources, summarized in Table 1. The framework of our study focuses on the context of OECD economies, encompassing data collected from 27 countries spanning the period from 1990 to 2020. Appendix Table 9 contains a list of the countries that are used for analysis for clarification. Based on the data that is readily available for the chosen countries, this particular timeframe and countries were chosen. The choice to focus on the OECD context is of paramount importance, as highlighted by Abid et al. (2022), who suggested that the examination of highly developed countries is crucial for safeguarding long-term global interests. The impacts generated by these economies transcend national boundaries and have far-reaching consequences that extend beyond their own territories.

**Table 1:** Data description

Variables	Abbreviation	Description	Sources
Environmental policy stringency	EPS	Environmental policy stringency index	OECD
Energy transition	ET	Renewable energy supply, % total energy supply	OECD
Climate change mitigation	CC	Carbon dioxide emissions (mt)	WDI
Gross domestic product	GDP	Gross domestic product (constant 2010 US$)	WDI
Non-renewable energy	NRE	Fossil fuel consumption (% of total)	WDI
Technological development	R&D	patent applications	WDI
Foreign direct investment	FDI	FDI net inflows (BoP, current US$)	WDI

#### Independent variables

Environmental policy stringency refers to the level of stringency in a country’s environmental policies, which can be measured in a manner that is both specific to the country and internationally comparable. Stringency, in this context, refers to the extent to which environmental policies establish a clear and tangible cost or value on activities that directly or indirectly contribute to pollution or harm the environment. This measurement helps assess how robust and effective a country’s environmental regulations are in promoting sustainable practices and discouraging detrimental behaviors (OECD 2021). By quantifying the stringency of environmental policies, we gain insights into the commitment and efforts made by OECD nations to address environmental challenges and foster a cleaner and greener future. The data is collected from OCED stats (OECD 2021).

Energy transition, commonly known as the shift toward renewable energy, signifies a global transition from predominantly fossil fuel-based energy systems to those primarily reliant on renewable energy sources. This transition’s primary goal is to address climate change by lowering greenhouse gas emissions and fostering environmentally friendly growth (Dong et al. 2022; Sun et al. 2023). The quantification of the energy transition is determined by calculating the proportion of renewable energy sources in comparison to the overall energy consumption as defined by Sun et al. (2023), and the data used for this measurement is obtained from OCED (2022).

#### Dependent variable

Climate change: The impact of climate change poses significant challenges, and the release of greenhouse gases (GHGs) is widely acknowledged as a critical global issue with far-reaching consequences for the entire ecosystem. Among these gases, carbon dioxide emissions are identified as the predominant factor contributing to anthropogenic GHG emissions. Previous studies have frequently employed CO_2_ emissions as a key indicator to assess the extent of environmental catastrophes (Abid et al. 2022; Chen et al. 2022). We have used CO_2_ as a representative of climate change, and the data for OECD countries is extracted from WDI (2021).

#### Control variables

As a control variable, we have used different environmental and non-environmental variables. Our first control variable is non-renewable energy consumption following Abid et al. (2023a). GDP (Mehmood 2021), FDI, and R&D (Abid et al. 2022; Fernández Fernández et al. 2018) are used as the non-environmental control variable, and data extracted from (US$ constant) (World Bank 2022).

### Slope heterogeneity

The slope homogeneity test, initially developed by Swamy (1970), is employed to assess whether the slope coefficients of the co-integration equation are homogeneous. This test examines the equality of the coefficients across different groups or subgroups within a panel dataset. By evaluating slope homogeneity, researchers can determine if the relationship between variables is consistent and comparable across different entities or time periods. The estimation of slope coefficients, whether they are homogeneous or not, is facilitated by the widely recognized Pesaran and Yamagata (2008) test of slope heterogeneity. This test builds upon the original Swamy (1970) test and offers refinements that enhance its reliability. Pesaran and Yamagata (2008) test provides both ordinary and adjusted measures of slope heterogeneity, making it a more robust and comprehensive approach. Compared to standard heterogeneity tests that do not account for cross-sectional dependency, Pesaran and Yamagata’s (2008) test addresses this issue effectively. It is particularly advantageous for analyzing datasets with short cross-sections and long periods, as the results from the standard test tend to be problematic in such cases.

The equations for Pesaran and Yamagata’s (2008) slope heterogeneity test are as follows:

1 $${\Delta}_{sh}={\left(\mathcal{N}\right)}^{\frac{1}{2}}I{(2k)}^{-\frac{1}{2}}+\left(\frac{1}{\mathcal{N}}s-k\right)$$

2 $${\Delta}_{\mathfrak{a} sh}=\left(\mathcal{N}I\ \left(\frac{2k{\left(\mathcal{T}-k-1\right)}^{-\frac{1}{2}}}{\mathcal{T}+1}\right)+\left(\frac{1}{\mathcal{N}}\mathcal{S}-k\right)\right)$$

*Δ*_*SH*_ and ${\Delta}_{\mathfrak{a} sh}$denote the adjusted slope coefficients of homogeneity. Furthermore, this indicates that the null supposition makes slope coefficients homogeneous, whereas the alternative hypothesis assumes heterogeneity.

### Cross-section and unit root test

To begin the data examination, we proceed to the cross-section dependence test. It is a vital step as it helps us assess the presence of interdependence among individual observations within the panel. It permits a broad consideration of the data structure and ensures the validity of subsequent econometric analyses. Detecting and treating cross-section dependence is essential because failing to account for it results in erratic parameter approximations, undermining the reliability of empirical findings (Flores 2019; Westerlund and Edgerton 2008). In doing so, we have used CSD tests designed by Breusch and Pagan (1980) and Pesaran (2007) tests, which are built on the statement that individual effects are cross-sectionally dependent. The test examines whether the individual-specific effects are correlated across cross-sectional units. Equations (1) and (2) represent Pesaran-CD and Breusch and Pagan-LM tests, respectively.345

Afterward, it becomes crucial to utilize second-generation unit root tests when analyzing panel data in order to address possible cross-sectional dependence. Failing to address this dependence can introduce bias and inconsistency in estimation results, compromising the reliability of subsequent statistical analyses (C. Li et al. 2023). In this study, we have utilized second-generation unit root tests, namely, the CADF and CIPS tests developed by Pesaran (2007), that enable the discovery of unit roots in panel data while considering cross-sectional dependence. These tests are specifically designed to detect unit roots in panel data. By incorporating lagged levels and first-difference terms of the dependent variable and other relevant variables, these tests effectively capture both the individual-specific effects and cross-sectional dependencies. Compared to first-generation tests, second-generation tests are more sensitive and superior in avoiding spurious results (Abid et al. 2023b; Ramzan et al. 2023). The CADF test equations are formulated as follows:6

The equation provided incorporates the coefficient *αi*, which signifies a deterministic trend. *K* represents the lag order, while *t* denotes time. Pesaran (2015) has proposed an approach to address the issue of cross-sectional dependence within the chosen sample. This method also produces consistent outcomes, even when data is limited. The CIPS test has gained recognition in recent studies due to its capability to effectively handle cross-sectional dependence and heterogeneity. The null hypothesis that needs to be rejected is the absence of a unit root in the sequence. If the variables exhibit stationarity at the first difference, performing a co-integration test before determining the parameters is recommended.

CIPS can be calculated with the average of CADF, and the equation will be as follows:7

By incorporating both lagged levels and first-difference terms of the variables, the CADF test can effectively capture both individual-specific effects and cross-sectional dependencies. This enhances the accuracy and reliability of unit root estimation in panel data analysis.

### Co-integration test

The next step in the research is to see if there is any integration between the factors being studied. Co-integration denotes a long-term relationship between the various elements in the model. We used the modern panel co-integration test presented by Westerlund and Edgerton (2008), which is specifically built for analyzing environmental policy, energy transition, and climate change scenarios to ensure trustworthy and robust results. The analysis focuses on determining if the error-correction term in a panel error-correction model is equal to 0. The co-integration test is mathematically represented as follows:8

The foregoing equations demonstrate the presence of co-integration between the variables. The coefficient ${\beta}_{\mathfrak{i}}$ represents the magnitude of the error correction term. The test equations can be shown as follows:8.18.2

8.3 $${P}_{\uptau}=\frac{a^{\prime }}{SE\left({}^{\prime}\right)}$$

8.4 $${P}_{\textrm{a}}=\frac{p_a}{T}$$

In above equations ${\mathcal{G}}_{\uptau}$ and ${\mathcal{G}}_{\mathfrak{a}}$ are group mean estimates and *P*_τ_ and ${P}_{\mathfrak{a}}$ represent panel estimates.

### Model construction

To investigate the relationship between environmental policy, energy transition, climate change, and control variables, we have chosen the CS-ARDL (common correlated effects autoregressive distributed lag) method as the primary research approach. This decision is driven by several factors, including the panel data nature of our dataset and the presence of cross-sectional dependency among the variables in our analysis. The CS-ARDL mechanism, originally proposed by Chudik et al. (2013), offers a robust framework for estimating long-run common coefficients for variables that exhibit intercountry dependency. In addition to estimating these coefficients, the CS-ARDL method also provides insights into the adjustment speed of the selected model. This approach effectively addresses challenges associated with CSD and heterogeneity, which are not adequately handled by traditional techniques such as FMOLS and DOLS. By incorporating dynamic common correlated impact predictors, the CS-ARDL method is able to effectively tackle the issues posed by heterogeneity and CSD, enhancing the accuracy and reliability of our analysis (Abid et al. 2023a; Li et al. 2022; Mehmood 2022). The CS-ARDL (commonly correlated augmented autoregressive distributed lag) method offers a significant advantage in handling panels with heterogeneous characteristics, where different units or individuals are observed over time. It effectively addresses individual-specific fixed effects, time-specific fixed effects, and the presence of common factors among the panel units. In a study by Narayan (2020), it is demonstrated that the CS-ARDL estimator possesses desirable properties such as asymptotic unbiasedness, consistency, and normal distribution, thereby establishing its reliability for panel data analysis. The author emphasizes its benefits, which include its capability to address cross-sectional dependence, individual-specific fixed effects, and endogeneity. The empirical evidence presented in the research paper provides support for the consistency and efficiency of the CS-ARDL estimator, further emphasizing its utility in panel data analysis. Furthermore, the ARDL approach is known to be more responsive than the cross-sectional distributed lag approach in determining the appropriate lag length.

The mathematical representation of the CS-ARDL model is as follows:9

Equation (9) introduces the autoregressive distributed lag model, while Eq. (10) represents its expanded version, which addresses the concern of inappropriate inference regarding the presence of threshold effects caused by CSD (Chudik and Pesaran 2015). To obtain Eq. (10), we average the cross-section of all the regressors involved in Eq. (9). This modification ensures that the issue of unfitting inference related to threshold effects is appropriately accounted for.10

The following are the average values of all the variables:11

Equation (10) incorporates the lagged variables *ax*, *az*, and *aw*, representing the previous values of the variables. In this equation, Hit denotes the per capita CO_2_ emissions, Zi.t represents the independent variables, and *X* represents the average cross-section, which accounts for spillover effects (Chandra et al. 2023). The CS-ARDL approach utilizes the short-run coefficient to predict the long-run coefficients. Equations (11) and (12) correspond to the mean group estimator and the long-run coefficients, respectively.1213

Short-run coefficients are14where $\Delta \mathfrak{l}=t-\left(t-1\right)$151617

The term “error correction term (ECT (− 1))” in the model indicates the presence and significance of the stability mechanism. This term captures the adjustment process that ensures the model returns to a long-term equilibrium after any short-term deviations.

## Results and discussion

Table 2 presents a concise overview of the standard descriptive statistical tests. The mean value of environmental policy stringency is calculated as 10.6186, indicating the average level of strictness or severity in environmental policies. Similarly, the mean value of energy transition is determined to be 2.2342, representing the average progress or advancement in transitioning to sustainable energy sources. On the other hand, non-renewable energy has an average value of 0.6738, signifying the typical amount or proportion of energy derived from non-renewable sources.

**Table 2:** Descriptive statistics

Variable	Mean	Median	SD	Min	Max
EPS	10.6186	11.1184	2.7618	4.7192	15.397
ET	2.2342	2.465	0.8468	1.2312	3.4108
CC	2.1454	2.115	1.3513	0.1225	5.6984
NRE	0.6738	0.6837	0.3814	0.4236	1.5686
GDP	8.3367	8.3252	1.7113	4.2768	11.1063
R&D	9.4851	9.3211	1.0386	7.5581	11.4264
FDI	1.5386	1.5818	0.5698	− 0.366	2.3506

Table 3 presents the outcomes of an examination conducted by Pesaran and Yamagata (2008) to assess the homogeneity of slopes. The presence of heterogeneity within the findings indicates a deviation from the assumption of fixed coefficients in the model. Instead, it suggests that the slope or coefficient varies across different countries, signifying potential variations in the relationship being analyzed. The identification of such heterogeneity raises concerns about the exactness and consistency of the model, highlighting the necessity for cautious interpretation of the results obtained from panel causality analysis, which relies on the assumption of homogeneity in the dependent variable. The results depicted in Table 3 provide empirical confirmation of the existence of heterogeneity in the slope parameters. Consequently, the relationship between variables may not exhibit consistency across all data points or individuals. Moreover, the statistical significance level of 1% indicates that this heterogeneity holds substantive meaning from a statistical standpoint.

**Table 3:** Slope heterogeneity

Statistics	Test and *P* value
$\overset{\sim }{\Delta }$	17.250** (0.000)
${\overset{\sim }{\Delta }}_{\textrm{adj}}$	19.375** (0.000)

** depicts significance at 1%

The subsequent phase entails evaluating cross-sectional dependence, which can be accomplished by employing various techniques. The findings are offered in Table 4. When cross-sectional dependence is detected, employing traditional econometric techniques (first-generation approaches) may yield misleading or erroneous results. Consequently, it becomes imperative to adopt second-generation approaches that consider the interdependence among cross-sections, thereby ensuring the acquisition of more dependable and valid outcomes.

**Table 4:** CD outcomes

Variables	Breusch-Pagan LM	Pesaran CD	Pesaran LM_adj_
EPS	1317.210**	38.5215**	14.287**
ET	2233.843**	61.1579**	18.692**
CC	1894.982**	74.8735**	9.858**
NRE	1690.865**	77.8980**	11.403**
GDP	1994.433**	81.4104**	10.384**
R&D	2162.073**	67.4136**	24.581**
FDI	1466.858**	52.4569**	15.412**

** indicates a 1% significance level

Subsequently, the outcomes of unit root are displayed in Table 5. The results indicate that all indicators, namely, EPS, ET, CC, NRE, GDP, and R&D, exhibit co-integration at the first difference.

**Table 5:** Unit root results

Variables	CIPS		CADF	
	I(0)	I(1)	I(0)	I(I)
EPS	− 2.494	−5.234***	− 2.159	− 3.620***
ET	− 2.307	− 2.992**	− 2.672	− 3.565***
CC	− 2.226	− 6.119***	− 1.868	− 4.321***
NRE	− 2.023	− 2.839**	− 2.492	− 2.975**
GDP	− 1.673	− 3.173***	− 2.123	− 3.010**
R&D	− 1.686	− 4.605***	− 1.157	− 4.839***
FDI	− 1.854	− 3.956***	− 1.779	− 2.915***

***, **, and * indicate 1%, 5%, and 10% significance levels

Co-integration signifies long-term connection or equilibrium among the variables. In this scenario, the aforementioned indicators demonstrate co-integration, implying a stable and consistent relationship among them, even though their individual behavior may exhibit short-term disparities. The identification of co-integration at the first difference suggests that changes or trends in these variables tend to move in harmony over time.

To inspect the co-integration among variables in the panel data, it is recommended to consider the order of integration, which signifies the number of differencing operations needed to achieve stationarity. In this study, an order of integration of *I*(1) is utilized, indicating the appropriateness of co-integration testing. Two co-integration tests are employed in this analysis. The first test utilized is the panel bootstrap co-integration test proposed by Westerlund and Edgerton (2008). The outcomes are displayed in Table 6, which indicates that the null hypotheses of co-integration are accepted. The Bootstrap *P*-values of the estimated Eqs. (1) and (2), both with trends and without trends, are found to be greater than 5% at the specified significance level. Regarding the estimated Eq. (3), co-integration is accepted for constants but rejected for the trend component.

**Table 6:** Westerlund cointegration results

Statistics	Value	*Z*-value	*P*-value
Gt	− 5.32	− 3.17	0.02
Ga	− 9.27	− 5.91	0.00
Pt	− 11.39	− 4.74	0.00
Pa	− 15.19	− 8.15	0.00

*** indicates a 1% significance level

Table 7 presents the outcomes of the CS-ARDL analysis, which provide insight into the relationship between main variables, with a particular emphasis on the impact on CO_2_ emissions. Findings stated a negative association between environmental policy and CO_2_ emissions, meaning that CO_2_ emissions decline as environmental program rigidity increases. Based on the coefficient of − 0.098, it can be inferred that a 1% rise in environmental policy strictness leads to a significant long-term decrease of 9.8% in CO_2_ emissions in OECD economies. The analysis underscores the significance of strengthening environmental policies in promoting environmental sustainability and mitigating pollution levels in advanced OECD economies. These findings are consistent with previous research conducted by Ahmed and Ahmed (2018) and Yirong (2022), emphasizing the positive impact of environmental policy stringency. The study by Albulescu et al. (2022) similarly discovered a substantial negative correlation between environmental policy stringency and CO_2_ emissions, indicating that stricter policies lead to greater reductions in emissions. Environmental policy stringency is important in its ability to impose explicit or implicit costs on activities that contribute to pollution or environmental harm. This approach incentivizes individuals, businesses, and industries to adopt cleaner and more sustainable practices, consequently yielding improved environmental outcomes.

**Table 7:** CS-ARDL analysis

Variables	Coefficients	*t*-statistics	*p*-values
Long-run coefficients			
EPS	− 0.098***	− 2.695	0.000
ET	− 0.134***	− 4.841	0.000
NRE	0.562***	8.462	0.000
GDP	0.160***	2.392	0.000
R&D	− 0.163***	− 4.920	0.000
FDI	0.629***	9.516	0.000
Short-run coefficients			
EPS	− 0.076*	− 1.862	0.000
ET	− 0.313	− 7.516	0.000
NRE	0.415***	7.410	0.000
GDP	0.073*	0.035	0.000
R&D	− 0.325**	− 4.920	0.000
FDI	− 0.148**	− 2.527	0.000

***, **, and * show significant levels at 1%, 5%, and 10%, respectively

The presence of negative coefficients in the analysis indicates that transitioning to renewable energy sources is an effective strategy for addressing climate change in both the short and long terms. The utilization of clean energy, such as renewables, plays a significant role in reducing greenhouse gas emissions, particularly CO_2_. The results are backed by Kabeyi and Olanrewaju (2022) and Niu et al. (2023). Renewable energy transitions from clean resources that can be utilized without compromising the energy requirements or climate conditions for future generations. The transition toward renewable energy presents the most effective solution for tackling the pressing challenge of climate change and reducing CO_2_ emissions. Fossil fuel combustion, a major contributor to greenhouse gas emissions, can be significantly mitigated through the adoption of renewable energy sources. Unlike fossil fuels, renewable energy generation produces minimal to no emissions during operation. By embracing renewable energy, we can substantially reduce our dependence on fossil fuels, curbing the release of CO_2_ into the atmosphere and thus combating climate change. Moreover, renewable energy sources such as solar, wind, and hydropower are abundant and readily available, providing sustainable alternatives that can meet our energy needs without compromising the climate or depleting resources for future generations. By transitioning to renewables, we can address the urgent environmental challenges and pave the way toward a cleaner and more sustainable future (Qin et al. 2021). A report by the International Renewable Energy Agency IRENA (2020) highlights the positive impact of renewable energy on CO_2_ emissions reduction. According to the IRENA study, the rapid deployment of renewable energy technologies has resulted in significant emission reductions. The study found that, on average, each additional megawatt-hour of renewable power capacity installed led to a reduction of about 0.5 metric tons of CO_2_ emissions in 2020.

The empirical evidence from studies by Abid et al. (2022), Al-mulali et al. (2015), and Nasir et al. (2019) indicates that both GDP and FDI have a significant positive impact on CO_2_ emissions in OECD countries. The relationship between GDP, FDI, and climate change is intricate yet interlinked. Economic growth, as measured by GDP, often leads to increased energy consumption and industrial activities, consequently resulting in higher levels of CO_2_ emissions. When countries actively seek foreign direct investment, they frequently prioritize industries that contribute to economic expansion but may also possess higher carbon footprints. The establishment of energy-intensive industries, the escalation of transportation activities, and the exploitation of natural resources are common outcomes of FDI, all of which can contribute to the emission of CO_2_. The pursuit of economic growth and the attraction of FDI, in the absence of proper environmental regulations and sustainable practices, can exacerbate climate change and environmental degradation. Therefore, it becomes crucial to strike a balance between economic development, foreign investment, and environmental sustainability in order to effectively address climate change and mitigate CO_2_ emissions.

The findings of studies conducted by Fernández Fernández et al. (2018) and Qin et al. (2021) indicate that R&D has a significant negative impact on CO_2_ emissions. R&D plays a pivotal role in the reduction of CO_2_ emissions by facilitating innovation and driving technological advancements. Through research and development efforts, innovative solutions such as carbon capture and storage, sustainable transportation systems, and energy-efficient buildings can be discovered and implemented. Ultimately, R&D contributes significantly to addressing climate change by providing the essential tools and knowledge required to transition toward a low-carbon economy and mitigate the adverse effects of CO_2_ emissions.

The results of the Comprehensive Cross-Entity Model Group (CCEMG) are presented in Table 8. The findings in the table affirm the substantial effect of EPS on climate change, as indicated by the negative coefficient value, which shows how crucial it is to have stringent policies in place to combat climate change in OECD. Furthermore, the negative values of energy transition (− 0.245) also demonstrate a significant negative impact on climate change, showing that energy transition is imperative to address environmental challenges and reduce CO_2_ emissions. In the case of NRE, GDP, and FDI, their respective coefficient values of 0.387, 0.445, and 0.173 indicate that these factors significantly contribute to the acceleration of climate change by increasing CO_2_ emissions in OECD countries. However, the negative coefficient value of R&D (− 0.154) suggests that increased research and development efforts can substantially reduce CO_2_ emissions in OECD countries, highlighting the importance of further investment in this area. Additionally, the robustness test supports the results obtained from our primary model.

**Table 8:** Robustness check

Dependent variable	CCEMG		
	Coefficients	*t*-statistics	*p*-values
EPS	− 0.142***	− 2.437	0.000
ET	− 0.245***	− 4.047	0.001
NRE	0.387**	10.791	0.004
GDP	0.445***	14.263	0.000
R&D	− 0.154**	− 3.854	0.000
FDI	0.173***	3.175	0.001

***, **, and * explain the significance level at 1%, 5%, and 10%, respectively

## Conclusion and policy implications

A hugely significant worldwide issue, climate change is principally caused by the buildup of greenhouse gases, especially carbon dioxide, in the Earth’s atmosphere. It has serious repercussions and is acknowledged as a major problem everywhere. In this study, the emphasis is on analyzing how environmental policy rigidity and the energy transition have affected climate change in OECD nations between 1990 and 2020. We have used thorough econometric methods to assess the short- and long-term correlations, incorporating control variables such non-renewable energy, FDI, R&D, and GDP. The results show that addressing climate change and reducing CO_2_ emissions require the adoption of strict environmental regulations and a shift to renewable energy sources (Ahmed and Ahmed 2018; Yirong 2022; Kabeyi and Olanrewaju 2022; Niu et al. 2023). In OECD nations, CO_2_ emissions are greatly increased by GDP and FDI. GDP-measured economic expansion results in higher levels of industrial activity and energy consumption, which raise CO_2_ emissions. Through transportation, resource exploitation, and energy-intensive businesses, FDI can also increase emissions (Abid et al. 2022; Al-mulali et al. 2015; Nasir et al. 2019). Unchecked economic expansion and FDI worsen environmental damage and climate change. To reduce CO_2_ emissions and combat climate change, a balance must be struck between development, investment, and sustainability (Abid et al. 2023b). CO_2_ emissions are negatively impacted by R&D. It encourages invention and technological development, making it possible to put into practice solutions like carbon capture, environmentally friendly transportation, and energy-efficient structures. For the transition to a low-carbon economy and to mitigate the negative consequences of CO_2_ emissions, R&D is crucial (Fernández Fernández et al. 2018; Qin et al. 2021).

The OECD nations, which include some of the most developed economies on earth, have a major impact on global CO2 emissions (Jebli et al. 2016). These countries continue to see an increase in emissions despite having made significant advancements in environmental policies and the switch to renewable energy sources. This increase can be linked to a number of factors, including industrial development, rising non-renewable energy use, and reliance on fossil fuels. Despite efforts to reduce emissions, economic growth and the requirements of contemporary lifestyles continue to put pressure on CO_2_ emissions in these nations. It is critical for OECD nations and the global community to prioritize sustainable development, make the transition to low-carbon economies, and adopt all-encompassing methods to cut greenhouse gas emissions in order to effectively address climate change (Wang et al. 2020). To address the challenge’s global scope, this entails supporting international cooperation, enforcing strict environmental rules, and investing in renewable energy. We can only hope to lessen the effects of climate change and ensure a sustainable future for humanity through collective efforts (Abid et al. 2023a). Finally, we used a robustness test to support the findings of our primary study model.

Based on the above, we have formulated the following policy suggestions. Firstly, The OECD should set ambitious emission reduction targets that align with the goals of the Paris Agreement, aiming to limit global temperature rise to well below 2 °C. These targets should be quantifiable, time bound, and consistently reviewed and updated to reflect the latest scientific evidence and technological advancements. By actively enhancing environmental policy stringency and implementing these policy recommendations, the OECD can significantly contribute to global efforts to reduce CO_2_ emissions. Through strong leadership and collaboration, the OECD can drive the transition toward a sustainable, low-carbon future and inspire other nations to adopt more stringent environmental policies. Secondly, promote and support the development and deployment of renewable energy technologies. The OECD should facilitate knowledge sharing, capacity building, and financial support to member countries to accelerate the transition toward renewable energy sources, such as solar, wind, and hydropower. Additionally, the OECD should encourage investment in research and development of breakthrough technologies in the renewable energy sector. The OECD should advocate for the adoption of stringent energy efficiency standards and regulations and provide guidance on best practices for improving energy efficiency. This can include promoting energy-efficient technologies, incentivizing energy audits and retrofits, and supporting innovation in energy-efficient solutions. Lastly, the OECD can effectively address the challenge of increasing CO_2_ emissions associated with economic growth by safeguarding economic goals. This can be done by promoting R&D to channel sustainable economic development, encouraging innovation in clean technologies, strengthening environmental policies and regulations, fostering resource efficiency by promoting renewable energy transition, and enhancing intellectual property rights protection. Such a comprehensive and balanced approach will help create a greener and more sustainable future for all OECD member countries and beyond.

The study mentioned has certain limitations that need to be acknowledged. Firstly, by solely focusing on environmental policies and renewable energy transitions, the study might not provide a comprehensive understanding of all the factors contributing to emissions. Furthermore, the study specifically focuses on OECD countries. While this selection allows for a focused analysis of countries with relatively similar socioeconomic contexts, policy frameworks, and energy profiles, it also limits the generalizability of the findings. Secondly, the sensitivity of the panel ARDL model to endogeneity and the potential bias arising from omitted variable issues are noteworthy. Moreover, the procedure of determining suitable lag structures for individual variables within the panel ARDL framework introduces complexity. The selection of optimal lag quantities holds significant importance in ensuring the credibility of outcomes. An inaccurate choice of lag numbers has the potential to introduce bias into coefficient estimates and consequently yield erroneous inferences. Thirdly, different regions and countries outside the OECD may have distinct socioeconomic contexts, diverse policy frameworks, and varied energy profiles that could lead to different outcomes. Therefore, caution should be exercised when attempting to extrapolate the results of the study to non-OECD countries. To address these limitations, future studies could explore a broader range of variables associated with climate change. By considering a diverse set of factors and variables in different contexts, researchers can gain a more comprehensive understanding of the challenges posed by climate change. Additionally, utilizing different methodologies and approaches can contribute to generating a wider range of insights and potential solutions. By employing a more comprehensive and varied approach, future research can help address the pressing challenge of climate change more effectively.

## Data Availability

The datasets used and/or analyzed during the current study are available from the corresponding author on reasonable request.

## Appendix

**Table:** Table 9

Country	
Australia	
Austria	
Belgium	
Canada	
Czech Republic	
Denmark	
Finland	
France	
Germany	
Greece	
Hungary	
Ireland	
Italy	
Japan	
Korea	
The Netherlands	
Norway	
Poland	
Portugal	
Slovak Republic	
Slovenia	
Spain	
Sweden	
Switzerland	
Turkey	
UK	
USA	
